# Oral health-related quality of life in children attending university special needs and paediatric dental clinics in Trinidad and Tobago: A parental perspective

**DOI:** 10.2340/aos.v84.43009

**Published:** 2025-02-27

**Authors:** Ramaa Balkaran, Satu Lahti, Visha Ramroop, Jorma I. Virtanen

**Affiliations:** a The University of the West Indies St. Augustine, Trinidad and Tobago; b Department of Community Dentistry, Institute of Dentistry, University of Turku, Turku, Finland; c Faculty of Medicine, University of Bergen, Bergen, Norway

**Keywords:** disabled children, oral health, parents, quality of life

## Abstract

**Objective:**

To analyse the Oral Health-Related Quality of Life (OHRQoL) of 6–18-year-old children visiting the special needs and paediatric dental clinics of the University of the West Indies (UWI).

**Material and methods:**

Parents/caregivers of all 6–18-year-old children (*n* = 201) attending the Special Needs Dental Clinic (SNDC) and Child Dental Health Clinic (CDHC) were recruited. The Oral Health Impact Profile (OHIP-14), shortened version, was employed. Outcomes calculated were severity, prevalence, and OHIP-14 domains. Chi-square and Mann-Whitney *U* tests, and logistic regression models served for statistical analyses.

**Results:**

Parents/caregivers of children with disabilities (*n* = 101) and without (*n* = 100) participated. The mean age of the children was 10.6 (3.4 standard deviation [SD]) from the SNDC and 11.3 (2.8 SD) from the CDHC. The OHRQoL differed slightly between mean severity scores for children with disabilities (6.41 ± 9.09) and without (7.01 ± 6.87) (*p* = 0.020). When adjusted for confounders, OHIP-14 impacts perceived occasionally, daily or very often, children with disabilities had poorer OHRQoL.

**Conclusions:**

The overall OHRQoL among the children visiting the UWI dental clinics was poor. The OHRQoL was poorer in children with disabilities in terms of oral impacts perceived occasionally, daily or very often. Children attending for ‘pain and filling’ had higher odds of having OHIP-14 impact than others.

## Introduction

Poor oral health is a common problem associated with patients with special needs worldwide. Persons with special needs have general conditions that may cause problems in both their general and oral health, and impact their quality of life (QoL) [1]. Their poorer QoL may be related to their difficulty in chewing and maintenance of their oral hygiene; there appears to be a higher prevalence of dental caries in their permanent dentition [2]. This population may delay seeking care due to inadequate support and a lack of tailored care programmes that address their specific needs. Further, they may be unable to differentiate oral health issues from other health related ones, ultimately leading to dental extractions when dental care is sought late [3].

Oral health is more than just the absence of oral diseases, and World Health Organization (WHO) defines it as ‘the state of the mouth, teeth and orofacial structures that enables individuals to perform essential functions such as eating, breathing and speaking, and encompasses psychosocial dimensions such as self-confidence, well-being and the ability to socialize and work without pain, discomfort and embarrassment’ [4]. Oral health-related diseases negatively affect self-esteem and oral health-related quality of life (OHRQoL) [5, 6]. The concept of OHRQoL involves patient’s assessment of the impact of their oral health on various dimensions of their daily life [7]. These dimensions include, for example, impairment, functional limitation, pain, discomfort, disability and handicap concerning the effect of these oral health dimensions on their QoL [8].

In children with disabilities, such as Down Syndrome, oral health impacted various aspects of their lives [7]. Also, for those on the autism spectrum disorder, this disability significantly and negatively affected the OHRQoL of preschool children and their families more than those without autism [7]. In addition, in children with cleft lip and palate, the OHRQoL was significantly worse than those without, across all dimensions, specifically in their social well-being [8, 9]. Both children and adolescents with cerebral palsy (CP), experienced a greater negative impact on their OHRQoL than those without CP [10]. In addition, dental caries negatively impacted the OHRQoL of both populations regardless of disability [10].

Several OHRQoL tools have assessed the impacts of oral diseases among different age groups in the population. The Oral Health Impact Profile (OHIP) [11] is one such tool used for adults and recently in child and adolescent populations [12–14]. The validity and reliability of this tool are both satisfactory when applied to adolescent populations [14, 15]. Parents and caregivers of children and adolescents with special needs have reported the impact of oral diseases on the OHRQoL [16, 17]. However, more research is needed in this important area.

Trinidad & Tobago (T&T) is a twin-island nation consisting of a pleural society in the English-speaking Caribbean. In 2024, one-fifth of the population were children under the age of 15 years and T&T has a high prevalence of non-communicable diseases and risk factors [18]. In T&T, all health services, including dental care are available at no cost to the public based on general taxation of salaries, and privately. However, not all public health centres offer dental care nor are they equally equipped to offer the full dental services. The inequality in the provision of services may lead to inequalities in the OHRQoL, especially in more vulnerable groups such as children with disabilities. However, there is no information about the OHRQoL of children in T&T.

The general aim of this study was to analyse the OHRQoL of 6–18-year-old children with special needs and paediatric patients at the University of the West Indies (UWI) School of Dentistry. We analysed the parental views of OHRQoL in children visiting the Special Needs Dental Clinic (SNDC) and the Child Dental Health Clinic (CDHC).

## Materials and methods

A cross-sectional study was conducted at the UWI school of dentistry, involving two dental clinics with a paediatric population: the SNDC and the UWI CDHC. The study included consecutive parents/caregivers of all 6–18-year-old children with and without special needs (*n* = 201) attending the UWI SNDC and the CDHC during the period of data collection (July 2022 to February 2023). Parents/caregivers of children under 6 or over 18 years old were excluded. The questionnaire used a validated and standardised international survey instrument, the shortened version of the English version of OHIP-14. This included 14 questions concerning the frequency of oral adverse impacts in the following seven dimensions: functional limitation, physical pain, psychological discomfort, physical disability, psychological disability, social disability, and handicap [14, 19], with a 1-month reference period [20, 21] in this study to assess the OHRQoL.

The principal investigator administered these questionnaires face-to-face to the patients and their parents in the SNDC, and a calibrated researcher also administered these in the CDHC.

The responses were obtained from the parents/caregivers of the patients and children who were able to express themselves verbally. Since all respondents in the parents/caregivers group provided responses, this study reports the parents’ and caregivers’ answers and not those of the children. Written informed consent was obtained from the respondents who fit the inclusion criteria. Participants were not identified in any way.

### Outcome variables

The OHIP-14 used a 5-point Likert scale which ranged from ‘very often’ (1) to ‘never’ (5), and ‘I don’t know’ as a 6th option [19]. All responses were then coded with the numbers: 0 (=never), 1 (=hardly ever), 2 (=occasionally), 3 (=fairly often), and 4 (=very often) [19, 22]. Three outcome variables were then calculated: severity, prevalence, and seven dimensions of the OHIP-14 domains as described by Rantavuori et al. [22]. The OHRQoL severity score was calculated by summing the scores for each of the 14 items to give a minimum score of 0, indicating good oral health related QoL, and a maximum score of 56 indicating the poorest OHRQoL. The prevalence that is the percentage of people reporting at least one OHIP-14 impact was calculated at two different threshold levels: ‘occasionally’ or ‘fairly often’ or ‘very often’ (OFoVo) and ‘fairly often’ or ‘very often’ (FoVo) [22, 23]. This measure was given a code of 1 when one or more impacts were reported occurring at a specific threshold of OFoVo or FoVo, and a code of 0 if not reported. The value for each conceptual dimension of the seven OHIP-14 domains was then calculated by adding the two item values together [19]:

1. ‘Functional Limitation’: trouble pronouncing words, worsened taste;
2. ‘Physical Pain’: aching in mouth, discomfort eating food;
3. ‘Psychological Discomfort’: feeling self-conscious or feeling tense;
4. ‘Physical Disability’: interrupted meals or poor diet;
5. ‘Psychological Disability’: difficulty relaxing, embarrassment;
6. ‘Social Disability’: irritability, difficulty in doing usual jobs;
7. ‘Handicap’: life less satisfying, inability to function.

When calculating OHIP summary scores and threshold levels, participants with missing values for more than two OHIP items (either due to non-response or answering ‘don’t know’) were assessed for exclusion from the analysis. However, as there were no missing values and not more than two don’t know responses for the OHIP items, none were excluded.

Next, participants were omitted from the analysis if data were absent for both items within any of the seven OHIP-14 domains; there were four such participants. If one item within a domain was missing, but a response was provided for the other item, the missing value was imputed as 0. Consequently, when calculating severity scores, missing values were substituted with the sample mean derived from non-missing responses within the specific group for the relevant OHIP item (*n* = 201). The number of items recoded as missing varied from 6 to 16 per domain.

### Statistical analysis

The prevalence of OHIP-14 impacts, at both the FoVo and OFoVo thresholds, and variables such as demographics (gender, age, ethnicity), parent/guardian, reason for visit, dental attendance (last visit to the dentist), rating of child’s oral health were reported with frequencies and percentages. The severity and domain scores were reported using means, standard deviations (SDs), and ranges. Chi-square test and the Mann-Whitney *U* test were used in the statistical analyses. Two sets of logistic regression were fitted for prevalence outcome (dependent variable) at the FoVo and OFoVo thresholds with disability (yes = 1) as the independent variable (*n* = 197). Covariates were dichotomised as 0 and 1 were: Gender (male = 1), Age (6–12 = 1), Ethnicity (Afro-Caribbean = 1), Parent/guardian (Mother = 1), Reason for visit (Pain+ Filling = 1), Last visit to the dentist (Two years/less = Less than one year + One year + Two years = 1), Rating of child’s oral health (Excellent+ Very Good = 1). The odds ratios (ORs) with 95% confidence intervals (CIs) were used to assess the association between OHIP-14 and these variables. Data were analysed with Statistical Package for the Social Sciences (SPSS) version 26.

### Ethical considerations

The protocol for the study was approved by the University of the West Indies Ethics Committee (CREC-SA.1609/05/2022).

## Results

During the period of data collection, there were 101 responses collected on children with disabilities and 100 without disabilities. The most common disability was autism (21.9%), and among the children with disabilities 22.9% were non-verbal.

Among children with disabilities the mean age was 10.6 (3.4 SD) whereas among those without disabilities the mean age was 11.3 (2.8 SD). Most children (68.3%) with disabilities were male, while just over half (52.0%) without disabilities were females. Just under one-third (32.3%) of all children had never attended the dentist before this visit according to their parents/caregivers. The majority of first-time attenders were those without a disability (44.0%) while the corresponding percentage for those with a disability was 20.8%. For children with disabilities, the accompanying parent was the mother in 62.4% of the cases and 74.0% in children without disabilities.

Table 1 shows the OHIP-14 impacts at the FoVo threshold level. The responses from the parents/caregivers suggested that children with disabilities tended to have better OHRQoL than children without disabilities in terms of the prevalence of impacts occurring fairly or very often with the prevalence being 29.7% versus 42.0% respectively.

**Table 1:** Prevalence (%) * of OHIP-14 impacts occurring fairly often or very often (FoVo) in children ≤ 18 years of age visiting the Special Needs Dental Clinic and Child Dental Health Clinic (CDHC) in Trinidad and Tobago (*n* = 201).

Variables		Children in SNDC *n* = 101 (Valid %)		*p*-value † *n* = 201	Children in CDHC *n* = 100 (Valid %)		*p*-value † *n* = 201
**FoVo** Yes No	72 129	**FoVo** **Yes**	**FoVo** **No**		**FoVo** **Yes**	**FoVo** **No**	0.07
		30 (29.7)	71 (70.3)		42 (42.0)	58 (58.0)	
**Gender** Male	117	17 (24.6)	52 (75.4)	0.10	15 (31.3)	33 (68.8)	**0.04 †**
Female	84	13 (40.6)	19 (59.4)		27 (51.9)	25 (48.1)	
**Age** 6–12-year-olds	132	23 (31.9)	49 (68.1)	0.44	20 (33.3)	40 (66.7)	**0.03 †**
13–18-year-olds	69	7 (24.1)	22 (75.9)		22 (55.0)	18 (45.0)	
**Ethnicity**		10 (25.0)	30 (75.0)	0.60	13 (44.8)	16 (55.2)	0.46
Afro Caribbean	69						
Indo Caribbean	46	5 (27.8)	13 (72.2)		9 (32.1)	19 (67.9)	
Mixed	86	15 (34.9)	28 (65.1)		20 (46.5)	23 (53.5)	
**Parent/guardian**		24 (38.1)	39 (61.9)	**0.05**	33 (44.6)	41 (55.4)	**0.04 †**
Mother	137						
Father	34	2 (11.1)	16 (88.9)		5 (31.3)	11 (68.8)	
Caregiver	30	4 (20.0)	16 (80.0)		6 (60.0)	4 (40.0)	
**Reason for visit**		7 (58.3)	5 (41.7)	**0.01 †**	5 (83.3)	1 (16.7)	**0.01 †**
Pain	18						
Filling	28	4 (50.0)	4 (50.0)		9 (45.0)	11 (55.0)	
Cleaning	21	0 (0.0)	11 (100.0)		6 (60.0)	4 (40.0)	
Check-up	114	16 (24.6)	49 (75.4)		13 (26.5)	36 (73.5)	
Other	20	3 (60.0)	2 (40.0)		9 (60.0)	6 (40.0)	
**Last visit to the dentist**		19 (32.8)	39 (67.2)	0.69	18 (41.9)	25 (58.1)	0.66
Less than one year	101						
One year	7	2 (50.0)	2 (50.0)		2 (66.7)	1 (33.3)	
Two years	20	4 (26.7)	11 (73.3)		1 (20.0)	4 (80.0)	
Pain/emergency	8	1 (33.3)	2 (66.7)		3 (60.0)	2 (40.0)	
Never	65	4 (19.0)	17 (81.0)		18 (40.9)	26 (59.1)	
**Rating of child’s oral health**		0 (0.0)	5 (100.0)	**0.05**	1 (25.0)	3 (75.0)	0.31
Excellent	9						
Very Good	29	2 (16.7)	10 (83.3)		4 (23.5)	13 (76.5)	
Good	63	10 (32.3)	21 (67.7)		13 (40.6)	19 (59.4)	
Fair	75	11 (26.2)	31 (73.8)		16 (48.5)	17 (51.5)	
Poor	25	7 (63.6)	4 (36.4)		8 (57.1)	6 (42.9)	

* Percentage of subjects reporting at least one OHIP impact at the FoVo threshold.

† ***p*** < 0.05 for the (Chi-square test) between children with and without disabilities.

At the OFoVo threshold, the differences in the responses from the parents/caregivers were statistically significant and suggested that children with disabilities tended to have better OHRQoL than in those with children without disabilities in terms of prevalence of impacts occurring occasionally, fairly, or very often. These OHIP-14 impacts, showed a prevalence of 52.5% versus 74.0% respectively (Table 2).

**Table 2:** Prevalence (%) * of OHIP-14 impacts occurring Occasionally or Fairly Often or Very Often (OFoVo) in children ≤ 18 years of age visiting the Special Needs Dental Clinic (SNDC) and Child Dental Health Clinic (CDHC) in Trinidad and Tobago (*n* = 201).

Variables		Children in SNDC *n* = 101 (Valid %)		*p*-value † *n* = 201	Children in CDHU *n* = 100 (Valid %)		*p*-value † *n* = 201
Yes No	127 74	**OFoVo** **Yes**	**OFoVo** **No**		**OFoVo** **Yes**	**OFoVo** **No**	**0.001†**
		53 (52.5)	48 (47.5)		74 (74.0)	26 (26.0)	
**Gender** Male	117	35 (50.7)	34 (49.3)	0.61	15 (31.3)	33 (68.8)	0.25
Female	84	18 (56.3)	14 (43.8)		11 (21.2)	41 (78.8)	
**Age** 6–12-year-olds	132	39 (54.2)	33 (45.8)	0.59	39 (65.0)	21 (35.0)	**0.01 †**
13–18-year-olds	69	14 (48.3)	15 (51.7)		35 (87.5)	5 (12.5)	
**Ethnicity**		19 (47.5)	21 (52.5)	0.06	21 (72.4)	8 (27.6)	0.56
Afro Caribbean	69						
Indo Caribbean	46	14 (77.8)	4 (22.2)		19 (67.9)	9 (32.1)	
Mixed	86	20 (46.5)	23 (53.5)		34 (79.1)	9 (20.9)	
**Parent/guardian**		37 (58.7)	26 (41.3)	0.26	55 (74.3)	19 (25.7)	0.04
Mother	137						
Father	34	8 (44.4)	10 (55.6)		13 (81.3)	3 (18.8)	
Caregiver	30	8 (40.0)	12 (60.0)		6 (60.0)	4 (40.0)	
**Reason for visit**		12 (100.0)	0 (0.0)	**0.00 †**	**OFoVo** 6 (100.0)	0 (0.0)	0.06
Pain	18						
Filling	28	5 (62.5)	3 (37.5)		16 (80.0)	4 (20.0)	
Cleaning	21	3 (27.3)	8 (72.7)		9 (90.0)	1 (10.0)	
Check-up	114	29 (44.6)	36 (55.4)		30 (61.2)	19 (38.8)	
Other	20	4 (80.0)	1 (20.0)		13 (86.7)	2 (13.3)	
**Last visit to the dentist**		30 (51.7)	28 (48.3)	0.70	35 (81.4)	8 (18.6)	0.23
Less than one year	101						
One year	7	3 (75.0)	1 (25.0)		3 (100.0)	0 (0.0)	
Two years	20	9 (60.0)	6 (40.0)		4 (80.0)	1 (20.0)	
Pain/emergency	8	2 (66.7)	1 (33.3)		4 (80.0)	1 (20.0)	
Never	65	9 (42.9)	12 (57.1)		28 (63.6)	16 (36.4)	
**Rating of child’s oral health**							
Excellent	9	0 (0.0)	5 (100.0)	**0.05**	3 (75.0)	1 (25.0)	0.51
Very Good	29	4 (33.3)	8 (66.7)		11 (64.7)	6 (35.3)	
Good	63	17 (54.8)	14 (45.2)		22 (68.8)	10 (31.3)	
Fair	75	24 (57.1)	18 (42.9)		28 (84.8)	5 (15.2)	
Poor	25	8 (72.7)	3 (27.3)		10 (71.4)	4 (28.6)	

* Percentage of subjects reporting at least one OHIP impact at the FoVo threshold.

† ***p*** < 0.05 for the (Chi-square test) between children with and without disabilities.

The mean scores for severity reported by the parents/caregivers which also included the impacts experienced hardly ever, imply that the OHRQoL differed very slightly but statistically significantly between the children with disabilities (6.41 ± 9.09) and those without (7.01 ± 6.87) (*p* = 0.020). Nevertheless, the maximum severity score was 41 (median: 2.0; range: 0–41) in participants of children with disabilities and 33 (median: 5.8; range: 0–33) in those without.

In addition, the responses given by parents/caregivers about their children with disabilities, suggested that they tended to have poorer OHRQoL impacts than those with children without disabilities in the domains of ‘Physical pain’, ‘Physical disability’, ‘Social disability’ and ‘Handicap’, but better OHRQoL in the domains of ‘Psychological disability’ and ‘Psychological discomfort’ than children without disability. However, the difference was only statistically significant for the ‘Psychological discomfort’ domain (Table 3).

**Table 3:** OHIP-14 values and range of dimensions comparing children visiting the Special Needs Dental Clinic (SNDC) and Child Dental Health Clinic (CDHC) in Trinidad and Tobago (*n* = 197).

Dimension	Mean ± SD SNDC [97] (Range)	Mean ± SD CDHC [100] (Range)	Mean ± SD Total [197] (Range)	p *
Functional Limitation	0.45 (±1.16) (0–6)	0.46 (±1.14) (0–7)	0.46 (±1.15) (0–7)	0.616
Physical Pain	1.84 (±2.67) (0–8)	1.74 (±2.04) (0–8)	1.79 (±2.37) (0–8)	0.334
Psychological Discomfort	0.99 (±1.72) (0–8)	2.09 (±2.30) (0–8)	1.55 (±2.10) (0–8)	0.000 *
Physical Disability	1.00 (±1.99) (0–8)	0.75 (±1.35) (0–7)	0.87 (±1.70) (0–8)	0.745
Psychological Disability	0.80 (±1.50) (0–7)	1.10 (±1.54) (0–6)	0.95 (±1.52) (0–7)	0.056
Social Disability	0.73 (±1.50) (0–8)	0.40 (±0.91) (0–4)	0.56 (±1.25) (0–8)	0.184
Handicap	0.46 (±1.29) (0–7)	0.35 (±1.03) (0–6)	0.41 (±1.16) (0–7)	0.612

* *p* < 0.05 (Mann-Whitney U Test)

When adjusted for gender, age, ethnicity, parent/caregiver, ‘reason for visit’, ‘last visit to the dentist’, ‘rating of child’s oral health’, the OHRQoL did not differ between children with and without disability at the FoVo threshold. The variables gender, parent/caregiver, ‘reason for visit’, and ‘rating of child’s oral health’ had greater effect on the OHRQoL than disability. Males had half the odds of having OHIP-14 impact, indicating better OHRQoL, than females. Mothers were more likely to report their child having OHIP-14 impacts than the fathers or guardians. Those attending for ‘pain and filling’ had higher odds of having OHIP-14 impact than those attending for ‘cleaning, check-up and other’, and those whose oral health was rated ‘excellent and very good’ had lower odds of having OHIP-14 impact than those with ‘good, fair and poor’ ratings (Table 4).

**Table 4:** Logistic regression analysis among parents of children ≤ 18 years of age visiting the Special Needs Clinic (SNDC) and Child Dental Health Clinic (CDHC) in Trinidad and Tobago at the FoVo threshold (*n* = 197).

Variables	B	SE	Wald	df	OR	95% CI	p *
Gender	-0.7	0.3	4.4	1	0.5	0.3–1.0	0.037 *
Age	-0.4	0.3	1.4	1	0.7	0.3–1.3	0.236
Disability	0.3	0.3	0.8	1	1.4	0.7–2.7	0.377
Ethnicity	0.01	0.3	0.001	1	1.0	0.5–2.0	0.971
Parent/guardian	0.9	0.4	5.3	1	2.4	1.1–4.9	0.022 *
Reason for visit	0.8	0.4	4.8	1	2.2	1.1–4.7	0.027 *
Last visit to the dentist	-0.007	0.3	0.000	1	1.0	0.5–2.0	0.985
Rating of child’s oral health	-1.0	0.5	4.6	1	0.4	0.1–0.9	0.032 *
Constant	-0.7	0.6	1.6	1	0.5		0.207

‘Prevalence’ of Oral Health Impact Profile (OHIP)-14 impacts was the dependent variable (answering fairly often or very often to one or more items = 1). Independent variables were dichotomised as 0 and 1were: gender (male = 1) Age (6–12 = 1), Disability (yes = 1), Ethnicity (Afro-Caribbean = 1), Parent/ guardian (Mother = 1), Reason for visit (Pain +Filling = 1) (Cleaning +Check-up + Other = 0), Last visit to the dentist (Two years/less = Less than one year + One year + Two years = 1) (Pain/ Emergency+ Never = 0), Rating of child’s oral health (Excellent+ Very Good = 1) (Good + Fair+ Poor = 0).

SE: standard error; df: degree of freedom; OR: odds ratio; CI: confidence interval.

* ***p*** < 0.05

Conversely, when adjusted for gender, age, ethnicity, parent/caregiver, reason for visit, last visit to the dentist, rating of child’s oral health the OHRQoL at the OFoVo threshold, children with disabilities had poorer OHRQoL. Children with a disability had 2.9 times higher odds of having OHIP-14 impact, indicating poorer OHRQoL, than children without a disability. In addition, ‘reason for visit’ had an even greater effect on OHRQoL, with those attending for ‘pain’ and ‘filling’ having had 3.9 times odds of having OHIP-14 impact than those attending for ‘cleaning, check-up and other’ (Table 5).

**Table 5:** Logistic regression analysis among parents of children ≤ 18 years of age visiting the Special Needs Clinic (SNDC) and Child Dental Health Clinic (CHDC) in Trinidad and Tobago at the OFoVo threshold (*n* = 197).

Variables	B	SE	Wald	df	OR	95% CI	p *
Gender	-0.2	0.3	0.3	1	0.8	0.4–1.6	0.600
Age	-0.5	0.4	2.4	1	0.6	0.3–1.2	0.125
Disability	1.1	0.4	8.9	1	2.9	1.4–5.9	0.003 *
Ethnicity	-0.07	0.3	0.05	1	0.8	0.5–1.8	0.930
Parent/guardian	0.4	0.3	1.5	1	1.5	0.8–3.1	0.215
Reason for visit	1.4	0.5	8.4	1	3.9	1.5–9.7	0.004 *
Last visit to the dentist	0.6	0.4	2.5	1	1.7	0.9–3.5	0.114
Rating of child’s oral health	-0.7	0.4	3.1	1	0.5	0.2–1.1	0.078
Constant	-0.2	0.6	0.1	1	0.8		0.706

‘Prevalence’ of Oral Health Impact Profile (OHIP)-14 impacts was the dependent variable (answering occasionally, fairly often or very often to one or more items = 1). Independent variables were dichotomized as 0 and 1were: gender (male = 1) Age (6–12 = 1), Disability (yes = 1), Ethnicity (Afro-Caribbean = 1), Parent/guardian (Mother = 1), Reason for visit (Pain+ Filling = 1) (Cleaning +Check-up+ Other = 0), Last visit to the dentist (Two years/less = Less than one year + One year + Two years = 1) (Pain/Emergency+ Never = 0), Rating of child’s oral health (Excellent+ Very Good = 1) (Good + Fair+ Poor = 0).

SE: standard error; df; degree of freedom; OR: odds ratio; CI: confidence interval.

* ***p*** < 0.05

## Discussion

Our cross-sectional study reports the OHRQoL of children with and without disabilities visiting the UWI CDHC and SNDC. The OHRQoL of children with disabilities and without disabilities was poor in this population of children as reported by their parents/caregivers. The OHRQoL was poorer in children with disabilities than in those without disabilities in terms of oral impacts perceived occasionally, daily, or very often. These findings were similar to a Brazilian study [1] in which a negative impact on their children’s OHRQoL was reported by caregivers of children with special needs. In a recent study in China, children with autism had a lower OHRQoL compared to those without autism [24]. A study from South Africa, on the caregivers’ perceptions using the Parent-Caregiver Perception Questionnaire (P-CPQ) showed a mean score of 12.9, which was a low score, given the range of the questionnaire from 0 to 64 [17]. Furthermore, parents of children with disabilities are often stressed and inundated by the behavioural challenges associated with caring for children with disabilities, especially when they lack coping skills and support [25].

### Children with disabilities

Generally poor oral health status and high caries prevalence have been shown to negatively affect the OHRQoL of children with special needs [26], while improvements in OHRQoL have resulted following oral treatment in this population [7]. There were more males than females in our study, possibly due to boys being genetically more disposed to certain disabilities than girls, such as in autism [27]. The latter was the most common disability reported in this population. Interestingly, the findings were statistically significant at the FoVo threshold with males having half the odds of experiencing OHIP-14 impact than females. The parents of children with disabilities may have been dealing with other serious medical conditions and either overlooked or underestimated the impact of oral health issues on their children. There may also have been a lack of awareness concerning the distinct oral health problems in their children because of the disability [26]. For instance, the children’s expression to pain, may have been delayed than the general population, this is commonly seen in children with Down Syndrome [28]. In addition, they may not be perceived, or may not experience psychological impacts, if their cognitive function is impaired, in the same way as those without a disability.

The private practitioner has an important role to play in the treatment of this population. Although it may be challenging for dentists, due to longer appointment times required to use behaviour management techniques; a lack of access to dental care by persons with disabilities can increase their unmet needs. The key role of both paediatric dentists and general dentists cannot be underestimated in transitioning these patients to adult dental care.

### Paediatric patients

The majority of children who had never visited a dentist before, were from the CDHC. This delayed attendance may have led to untreated oral problems, pain and infection, and negatively impacted their oral health and overall well-being. In addition, when adjusted for other variables in the bivariate analyses, the children without disabilities had poorer OHRQoL. Irregular attendees frequently reported a poor rating of the OHIP-14 [29]. Carious lesions, especially when left untreated have been shown to be a primary factor negatively affecting the QoL in children [30]. Also, there were more adolescent children attending the CDHC; and research has shown that perceptions of OHRQoL issues may quickly change with the physical and psychosocial development of a child [31]. In this study, the models were adjusted for age. In addition, the psychological impact of OHRQoL tends to increase with age as patients become more mindful about the significance of their oral health [32]. This could have accounted for the worse mean OHRQoL at the domains of ‘Psychological disability’ and ‘Psychological discomfort’ in the children attending the CDHC.

### Comparison between the two patient groups

Children with disabilities had poorer OHRQoL in the domains of ‘Physical pain’, ‘Physical disability’, ‘Social disability’, and ‘Handicap’. However at the FoVo and OFoVo thresholds, children without disabilities had poorer OHRQoL. At the time of this research, there was a cost attached to all treatment for children at the CDHC whereas all the treatment costs for children attending the SNDC were covered by a grant from the Community Chest Limited. This could have accounted for the difference in more responses that included the ‘occasionally’ response at the OFoVo threshold which showed the reported OHRQoL was better in those children with disabilities at this threshold and was statistically significant.

The cost may have resulted in this delayed attendance until children from the CDHC were symptomatic and could have resulted in a selection of patients based on cost and ability to access oral care at the onset of symptoms. Socioeconomic factors may also have impacted on attendance which is important since these have been associated with poorer OHRQoL in children [30]. There may also have been a lack of knowledge, by the parents and caregivers, on the significance of oral health in relation to general health [17]. This may have contributed to the delayed attendance seen in children attending the CDHC, since their parents and caregivers are responsible for both identifying their oral health need and facilitating their dental visit.

In this study, there were more mothers who attended with children without disabilities. When adjusted for other variables, mothers reported their children having higher odds of experiencing OHIP-14 impacts daily or very often compared to fathers and other caregivers. Since mothers have been shown to be better proxies in children with special health care needs, this may have impacted on the responses in those with disabilities [33]. Furthermore, dental caries may significantly and negatively impact the OHRQoL [10]. It can also affect several dimensions of OHRQoL, including functional limitation, physical pain, psychological discomfort, and physical disability. This study did not assess the caries experience of the population; therefore, we are unable to conclude whether the lower OHRQoL was attributable to oral health status or the disabilities themselves. However, in 2019 just less than one-third of the child population had untreated caries of both in the deciduous teeth and permanent teeth [34]. Thus, children with higher caries experience compared to those without may have accounted for the poorer OHRQoL observed.

### National aspects

In T&T, oral care is available at clinics through private, government, and the UWI dental school. In government clinics, oral care is provided by dental nurses for children from age 2 to 12; and includes preventive, restorative, and extraction services [35]. Dental treatment for children over 12, is provided by dentists, and is often limited to extractions. There is a long wait time at government clinics which do not offer services to children on a daily basis. In addition, for those with special needs; the UWI SNDC is the only one of its kind, that offers specialised services to this population of patients at a reduced or no cost. These factors combined with the generally high costs of private dental care may have accounted for the high utilisation of services at the UWI dental clinics for children.

Affordability for dental care is a major issue in T&T, since most dental services are accessed through private practitioners in the Caribbean nation. The Oral Health Survey of school children in T&T, conducted in 2004, showed a higher Decayed, Missing, Filled Teeth (DMFT) score in the 6–8-year-olds versus the 12- and 15-year-olds [36]. Accessibility may also have played a part in the children not visiting a dentist previously, since the there are few dentists with 15 at public dental clinics and the other 258 in private practices in 2006 [36].

## Strengths and limitations

This study is the first of its kind in this population and demonstrated the OHRQoL among the two groups of children visiting the university clinics. The groups of children were compared and possible confounders were adjusted for in the logistic models. In addition, this research used experienced and calibrated examiners to administer the questionnaires with a reliable methodology based on similar research in this field. Furthermore, the responses from the parents and children were compared, and they showed alignment in the OHRQoL. Similar agreement on OHRQoL between children and their parents at a group level based on the total scores have been observed earlier [37]. The inclusion of the option ‘don’t know’ in the questionnaire ensured content validity by parents who did not have to presume a response or omit it altogether [37]. Although using parents as proxies in this study was necessary with many non-verbal children, this is a limitation since their knowledge about their children’s subjective emotional and social experiences, particularly when away from their parents, may not have been fully encapsulated in these responses [37]. Given the cross-sectional design of this study, these results cannot be used to determine the cause-and-effect relationships or be generalised.

Given that preventive approaches and regular oral health examinations may maintain good OHRQoL [6], we propose regular dental attendance in rural and urban communities with comprehensive treatment. This would be empowered by comprehensive oral health education for parents and caregivers, and the removal of cost for comprehensive dental care to all children, especially those with special needs. These can be funded through government and community programmes.

## Conclusions

This research highlighted the OHRQoL of children with and without special needs in T&T. The overall OHRQoL was poor, especially in those with special needs. However, at the both FoVo and OFoVo thresholds and domains: ‘Psychological Discomfort’ and ‘Psychological Disability’; children without disabilities in this population had worse OHIP-14 impacts. Delayed attendance and cost may have influenced these results.

## Data Availability

The datasets used and/or analysed during the current study are available from the corresponding author on reasonable request.

## References

[1] Cancio V, Faker K, Bendo CB, Paiva SM, Tostes MA. Individuals with special needs and their families’ oral health-related quality of life. Braz Oral Res. 2018;32:e39. 10.1590/1807-3107bor-2018.vol32.0039 29846384

[2] Pecci-Lloret MR, Pecci-Lloret MP, Rodríguez-Lozano FJ. Special care patients and caries prevalence in permanent dentition: a systematic review. Int J Environ Res Public Health. 2022;19(22):15194. 10.3390/ijerph192215194 36429911 PMC9690089

[3] Denis F, Becquet H, Renaud M, Savard G. Promoting oral health for patients with special needs. Int J Environ Res Public Health. 2023;20(13):6232. 10.3390/ijerph20136232 37444080 PMC10341822

[4] World Health Organization Oral Health [Internet]. 2023 [cited 2024 Dec 16]. Available from: https://www.who.int/health-topics/oral-health

[5] Sheiham A. Oral health, general health and quality of life. Bull World Health Organ. 2005;83(9):644–4. 16211151 PMC2626333

[6] Omara M, Stamm T, Bekes K. Four-dimensional oral health-related quality of life impact in children: a systematic review. J Oral Rehabil. 2021;48(3):293–304. 10.1111/joor.13066 32757443 PMC7984176

[7] Aljameel HA. Oral Health-related quality of life outcomes for individuals with disabilities: a review. J Clin Diagn Res. 2020;14(10):JE01–6. 10.7860/JCDR/2020/46100.14097

[8] Locker D. Measuring oral health: a conceptual framework. Community Dent Health. 1988;1:3–18. 3285972

[9] Kortelainen T, Tolvanen M, Luoto A, Ylikontiola LP, Sándor GK, Lahti S. Comparison of oral health-related quality of life among schoolchildren with and without cleft lip and/or palate. Cleft Palate Craniofac J. 2016;53(5):e172–6. 10.1597/14-180 26171571

[10] de Castelo Branco Araújo T, Nogueira BR, Mendes RF, Júnior RRP. Oral health-related quality of life in children and adolescents with cerebral palsy: paired cross-sectional study. Eur Arch Paediatr Dent. 2022;23(3):391–8. 10.1007/s40368-022-00694-x 35124753

[11] Slade GD, Spencer AJ. Development and evaluation of the oral health impact profile. Community Dent Health. 1994;11:3–11. 8193981

[12] Giannetti L, Murri A, Vecci F, Gatto R. Dental avulsion: therapeutic protocols and oral health-related quality of life. Eur J Paediatr Dent. 2007;8(2):69–75. 17571930

[13] Montero J, Costa J, Bica I, Barrios R. Caries and quality of life in Portuguese adolescents: impact of diet and behavioural risk factors. J Clin Exp Dent. 2018;10(3):e218–23. 10.4317/jced.54469 29721221 PMC5923895

[14] Riva F, Seoane M, Reichenheim ME, Tsakos G, Celeste RK. Adult oral health-related quality of life instruments: a systematic review. Community Dent Oral Epidemiol. 2021;50(5):333–8. 10.1111/cdoe.12689 34409626

[15] Silveira MF, Pinho L, Brito MFSF. Validity and reliability of the oral health impact profile instrument (OHIP-14) in adolescents. Paidéia (RibeirãoPreto). 2019;29:e2921. 10.1590/1982-4327e2921

[16] da Silva ACF, Barbosa TS, Gavião MBD. Parental perception of the oral health-related quality of life of children and adolescents with autism spectrum disorder (ASD). Int J Environ Res Public Health. 2023;20(2):1151. 10.3390/ijerph20021151 36673908 PMC9859466

[17] Nqcobo C, Ralephenya T, Kolisa YM, Esan T, Yengopal V. Caregivers’ perceptions of the oral-health-related quality of life of children with special needs in Johannesburg, South Africa. Health SA. 2019;24:1056. 10.4102/hsag.v24i0.1056 31934405 PMC6917375

[18] Pan American Health Organization. Trinidad and Tobago Country Profile [Internet]. 2024 [cited 2024 Dec 16]. Available from: https://hia.paho.org/en/countries-22/trinidad-tobago-country-profile#situation

[19] Slade GD, Nuttall N, Sanders AE, Steele JG, Allen PF, Lahti S. Impacts of oral disorders in the United Kingdom and Australia. Br Dent J. 2005;198(8):489–93. 10.1038/sj.bdj.4812252 15849587

[20] Slade GD. Derivation and validation of a short-form oral health impact profile. Community Dent Oral Epidemiol. 1997;25:284–90. 10.1111/j.1600-0528.1997.tb00941.x 9332805

[21] Sutinen S, Lahti S, Nuttall NM, Sanders AE, Steele JG, Allen PF, et al. Effect of a 1-month vs. a 12-month reference period on responses to the 14-item oral health impact profile. Eur J Oral Sci. 2007;115(3):246–9. 10.1111/j.1600-0722.2007.00442.x 17587301

[22] Rantavuori K, Silvola A-S, Suominen A, Masood M, Suominen AL, Lahti S. Gender differences in the association between malocclusion traits and oral health-related quality of life in Finnish adults. Eur J Oral Sci. 2023;131(3):e12927. 10.1111/eos.12927 36855237

[23] Raittio E, Lahti S, Kiiskinen U, Helminen S, Aromaa A, Suominen AL. Inequality in oral health-related quality of life before and after a major subsidization reform. Eur J Oral Sci 2015;123:267–75. 10.1111/eos.12192 26015152

[24] Du RY, Yiu CKY, King NM. Health- and oral health-related quality of life among preschool children with autism spectrum disorders. Eur Arch Paediatr Dent. 2020;21:363–71. 10.1007/s40368-019-00500-1 31802429

[25] Hsiao Y. Parental stress in families of children with disabilities. Interven Sch Clin. 2018;53(4):201–5. 10.1177/1053451217712956

[26] Puthiyapurayil J, Anupam Kumar TV, Syriac G, Maneesha R, Raseena KT, Najmunnisa. Parental perception of oral health related quality of life and barriers to access dental care among children with intellectual needs in Kottayam, central Kerala – a cross sectional study. Spec Care Dentist. 2022;42(2):177–86. 10.1111/scd.12658 34614254

[27] Werling DM, Geschwind DH. Sex differences in autism spectrum disorders. Curr Opin Neurol. 2013;26(2):146–53. 10.1097/WCO.0b013e32835ee548 23406909 PMC4164392

[28] Hennequin M, Morin C, Feine JS. Pain expression and stimulus localisation in individuals with Down’s syndrome. Lancet. 2000;356(9245):1882–7. Erratum in: Lancet. 2001;357(9259):890. 10.1016/S0140-6736(00)03259-1 11130384

[29] Kaprio H, Suominen AL, Lahti S. Association between subjective oral health and regularity of service use. Eur J Oral Sci. 2012;120:212–17. 10.1111/j.1600-0722.2012.00955.x 22607337

[30] Piovesan C, Antunes JL, Guedes RS, Ardenghi TM. Impact of socioeconomic and clinical factors on child oral health-related quality of life (COHRQoL). Qual Life Res. 2010;19(9):1359–66. 10.1007/s11136-010-9692-7 20571918

[31] Hettiarachchi RM, Kularatna S, Byrnes J, Scuffham PA. Pediatric quality of life instruments in oral health research: a systematic review. Value Health. 2019;22(1):129–35. 10.1016/j.jval.2018.06.019 30661626

[32] De Stefani A, Bruno G, Irlandese G, Barone M, Costa G, Gracco A. Oral health-related quality of life in children using the child perception questionnaire CPQ11–14: a review. Eur Arch Paediatr Dent. 2019;20(5):425–30. 10.1007/s40368-019-00418-8 30762210

[33] Alwattban RR, Alkhudhayr LS, Al-Haj Ali SN, Farah RI. Oral health-related quality-of-life according to dental caries severity, body mass index and sociodemographic indicators in children with special health care needs. J Clin Med. 2021;10(21):4811. 10.3390/jcm10214811 34768328 PMC8584947

[34] World Health Organization. Oral health country profile [Internet]. 2021 [cited 2024 Dec 16]. Available from: https://cdn.who.int/media/docs/default-source/country-profiles/oral-health/oral-health-tto-2022-country-profile.pdf?sfvrsn=f860371a_7

[35] Government of the Republic of Trinidad and Tobago, Ministry of Health. Dental services [Internet]. [cited 2024 Dec 16]. Available from: https://health.gov.tt/services/dental-services

[36] Naidu RS, Prevatt I, Simeon D. The oral health and treatment needs of schoolchildren in Trinidad and Tobago: findings of a national survey. Int J Paed Dent. 2006;16:412–8. 10.1111/j.1365-263X.2006.00755.x 17014539

[37] Jokovic A, Locker D, Guyatt G. How well do parents know their children? Implications for proxy reporting of child health-related quality of life. Qual Life Res. 2004;13:1297–307. 10.1023/B:QURE.0000037480.65972.eb 15473508

